# A Rare Case of Septic Ovarian Thrombophlebitis Caused by Tissierella praeacuta

**DOI:** 10.7759/cureus.42385

**Published:** 2023-07-24

**Authors:** Taruna Chandok, Sabirah N Kasule, Paul J Kelly, Efrain Gonzalez, Sridhar S Chilimuri, Cosmina B Zeana

**Affiliations:** 1 Internal Medicine, BronxCare Health System, Bronx, USA; 2 Infectious Disease, BronxCare Health System, Bronx, USA

**Keywords:** clostridium, tissierella, thrombophlebitis, ovarian vein thrombosis, gonadal vein thrombosis

## Abstract

We report a case of *Tissierella praeacuta* bacteremia and septic thrombophlebitis of the ovarian vein as a rare puerperal complication in a young patient. She was successfully managed with subcutaneous low molecular weight heparin (LMWH) and intravenous (IV) antibiotics before transitioning to a prolonged course of oral antibiotics at discharge.

## Introduction

Ovarian vein thrombosis (OVT) is an uncommon puerperal complication strongly associated with delivery by cesarean section and postpartum endometritis or chorioamnionitis [1]. Incidence is 0.05-0.16% of all vaginal births and up to 2% of cesarean sections [2]. *Tissierella praeacuta* is a rare human infection. Bacteremia is similarly uncommon and usually associated with invasive infections, including brain abscesses, osteomyelitis, and pyonephrosis [3]. A recent case report described septic pylephlebitis in a 67-year-old female who presented with fatigue and anorexia [4]. To our knowledge, our case is the second report of an endovascular infection with *T. praeacuta* and the first reported case of septic thrombophlebitis of the ovarian vein caused by *T. praeacuta*. Guidelines for the appropriate management of septic OVT are scant, so we hope to add to the greater body of literature on this rare diagnosis.

## Case presentation

A 24-year-old G2P2002 female presented six days after an uncomplicated vaginal delivery with fever, abdominal pain, nausea, and vomiting. The abdominal pain had begun in her bilateral lower quadrants shortly after delivery. Four days prior to admission, the pain had localized to the right lower quadrant (RLQ). Past medical history was significant for iron deficiency anemia and depression. 

On admission, she was afebrile and hemodynamically stable. Examination revealed tenderness to palpation and rebound in the RLQ, but no guarding. Laboratory tests were notable for leukocytosis of 15.6 k/µL (reference 4.8-10.8 k/µL), hemoglobin of 11 g/dL (reference 12.0-16.0 g/dL), sedimentation rate of 50.0 mm/h (reference <30 mm/h), C-reactive protein of 160.27 mg/L (reference <3.0 mg/L), aspartate aminotransferase of 142 U/L (reference 9-36 U/L), alanine aminotransferase of 80 U/L (reference 5-40 U/L), and alkaline phosphatase of 715 U/L (reference 43-160 U/L). Human immunodeficiency virus screening was negative. Contrast-enhanced computed tomography of the abdomen and pelvis showed expansion of the right ovarian vein with internal hypodensity, hyperemia of the mucosa, and surrounding inflammation suggestive of OVT extending from the right adnexa into the inferior vena cava. She was empirically started on subcutaneous low molecular weight heparin (LMWH) for the OVT and intravenous (IV) piperacillin-tazobactam for suspected endometritis.

**Figure 1:**
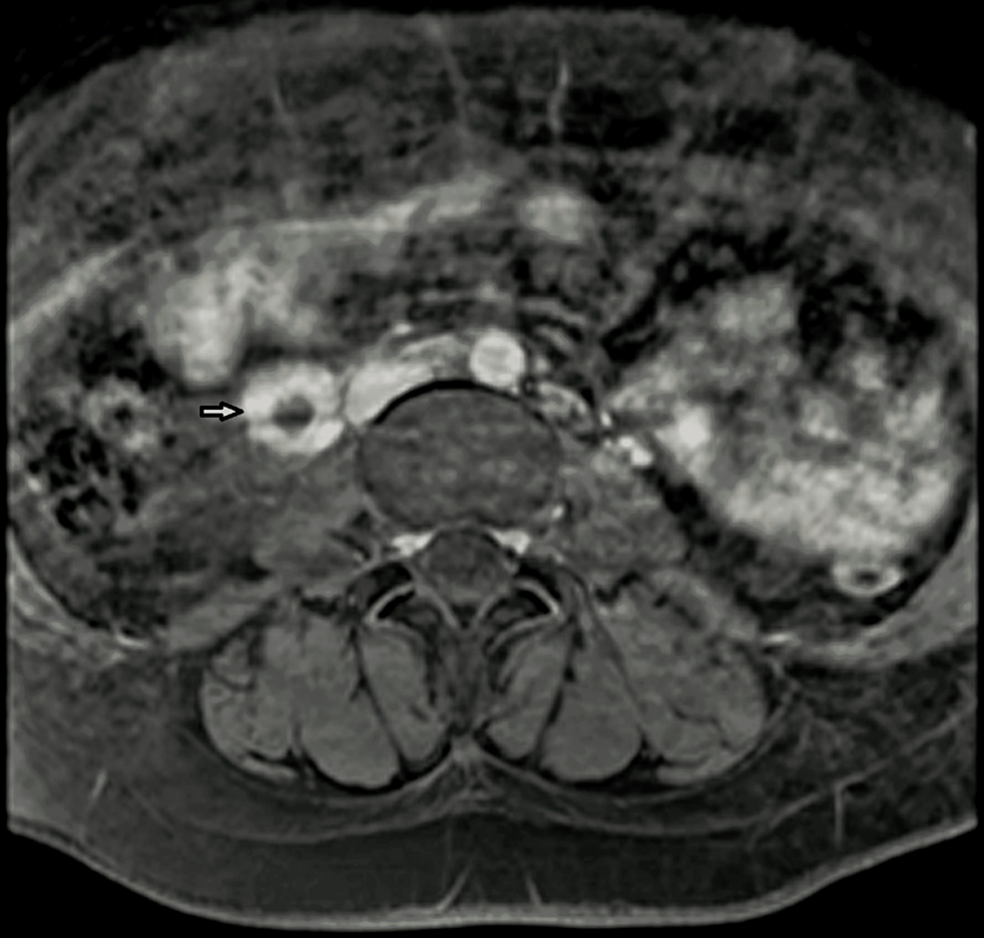
Right-sided thrombophlebitis is shown with an arrow; there is thickening of the gonadal vein with thrombus and surrounding edema.

One day after admission, the patient developed a fever of 103.1°F, and vancomycin was added for empiric methicillin-resistant *Staphylococcus aureus *(MRSA) coverage given her recent admission. On hospital day 3, Gram-variable bacilli were isolated from her admission anaerobic blood cultures, and on hospital day 6, these resulted as *T. praeacuta*. Her fevers persisted, so she was escalated to IV meropenem and clindamycin on hospital day 7. Vancomycin was discontinued. A follow-up retroperitoneal ultrasound noted a completely occluded, dilated right ovarian vein. Magnetic resonance imaging (MRI) of the abdomen and pelvis continued to show the thrombus, but there were no additional abdominal or pelvic complications (Figure 1). Vascular surgery deferred intervention.

Gradually, the patient’s transaminases improved, her leukocytosis resolved, and, on hospital day 9, she defervesced. Repeat blood cultures were negative. Unfortunately, our reference lab could not perform susceptibilities for *T. praeacuta*, so we relied on data from previous case reports, which showed susceptibility of *T. praeacuta* to beta-lactams, chloramphenicol, meropenem, and metronidazole [3]. Oral metronidazole was chosen at discharge for its excellent bioavailability and broad anaerobic coverage. Oral amoxicillin-clavulanate was also included to cover for *Streptococcus* species that may not have grown.

Two months after discharge, a repeat MRI showed interval resolution of her right OVT. At clinic follow-up two weeks after the MRI, she reported mild, cramping abdominal pain but was otherwise doing well. Blood cultures from this visit were negative. In total, she completed 10 days of IV antibiotics, four additional weeks of oral antibiotics, and three months of anticoagulation for septic OVT, achieving both symptomatic and radiologic resolution.

## Discussion

Puerperal OVT typically presents within a week of delivery with fever, leukocytosis, and lower flank or abdominal pain. The latter is usually right-sided, as 70-90% of OVT occurs in the right ovarian vein because it is longer and has multiple incompetent valves [5]. The pathogenesis of OVT is related to venous stasis and hypercoagulability, both of which are accentuated in pregnancy and further exacerbated by infection and/or cesarean section [6]. Infectious sequelae of OVT can include ovarian abscess, uterine necrosis, or septic thrombophlebitis [2]. Consistent with the literature, our patient was early postpartum, had a fever and leukocytosis, and her abdominal pain and OVT were right-sided. 

Anticoagulation and antibiotics are the foundation of noninvasive management of OVT [2]. The decision to treat, however, has come under review in recent years, especially as, occasionally, OVT can resolve on its own [7]. A 2017 retrospective review concluded that there was no statistically significant difference in outcomes between those who were and those who were not treated for OVT. They do comment that their review was limited by its retrospective nature, small sample size, and underpowered studies [2]. Septic OVT generally warrants treatment, though the diagnosis is challenging as bacteremia may be transient. In a 2006 review, less than 35% of OVT cases had positive blood cultures [8]. Therefore, a lack of bacteremia does not exclude a diagnosis of septic OVT and should not preclude antibiotics if the diagnosis is strongly suspected. Common microbes in septic OVT include streptococci, Enterobacteriaceae, and anaerobes [9]. Our patient’s empiric antibiotics were chosen to account for these pathogens, and her anticoagulation regimen was modeled after that for deep vein thrombosis.

*Tissierella praeacuta *is an obligately anaerobic, Gram-positive bacillus originally isolated from infant feces by Tissier in 1908 [10]. It shares biochemical profiles, 16S ribosomal ribonucleic acid (rRNA) gene sequences, and 96.5% of its deoxyribonucleic acid (DNA) with* Clostridium hastiforme*, which was named in 1939 by Maclennan [11]. Today, *T. praeacuta *and *C. hastiforme *are considered the same species [12]. Although classified as a Gram-positive organism, the cells of certain *Tissierella* species may stain Gram-negative or Gram variable [13], confounding appropriate empiric therapy. Identification can require matrix-assisted laser desorption/ionization-time of flight mass spectrometry (MALDI-TOF MS) or 16S rRNA sequencing [14]. Neither was required in our case, but they could have accelerated identification and reduced our patient’s length of stay.

## Conclusions

Decisions to treat puerperal OVT are complex, but in the right patient, particularly one with septic OVT, treatment can lead to successful outcomes. Practitioners should be aware that a Gram-negative or Gram-variable organism growing in a blood culture in this setting may actually be *T. praeacuta*, which is Gram-positive. Empiric coverage, therefore, should include antibiotics with broad anaerobic coverage, including metronidazole or current beta-lactam/beta-lactamase inhibitor combinations. We favor prolonged courses of treatment, which can be oral as long as the antibiotic has good oral availability. Anticoagulation choice and duration should be guided by hematology. 
